# Eye Washing Downregulated Angiotensin-Converting Enzyme 2 in Conjunctival Tissue Samples from Smokers

**DOI:** 10.3390/ijms242417526

**Published:** 2023-12-15

**Authors:** Hiroshi Fujishima, Hiroyuki Yazu, Eisuke Shimizu, Naoko Okada, Kazumi Fukagawa

**Affiliations:** 1 Department of Ophthalmology, Tsurumi University School of Dental Medicine, Yokohama 230-8501, Japan; g.h.yazu@gmail.com (H.Y.); nokada@nch.go.jp (N.O.); 2 Keio Allergy Center, Keio University School of Medicine, Tokyo 160-8582, Japan; ophthalmolog1st.acek39@keio.jp (E.S.); fukakazu0706@gmail.com (K.F.); 3 Department of Pharmaceutical Sciences, Nihon Pharmaceutical Hospital, Saitama 362-0806, Japan; 4 Ryogoku Eye Clinic, Tokyo 130-0026, Japan

**Keywords:** angiotensin-converting enzyme 2 (ACE2), transmembrane protease serine 2 (TMPRSS2), SARS-CoV-2 infection, smoking, eye rinsing

## Abstract

This study aimed to (1) determine whether the expression of angiotensin-converting enzyme 2 (ACE2) and transmembrane protease serine 2 is increased in tobacco smokers, which potentially increases their susceptibility to severe acute respiratory syndrome coronavirus 2 (SARS-CoV-2) infection, and (2) assess whether eye rinsing can reduce susceptibility. This prospective study included 20 eyes of 10 smokers and 18 eyes of nine healthy non-smokers (control) for reverse-transcription polymerase chain reaction. This study also included 28 eyes of 14 smokers and 16 eyes of eight healthy non-smokers (control) for enzyme-linked immunosorbent assay. Tear and impression cytology samples were collected from the right eye of each patient. The left eye was then rinsed for 30 s, and after 5 min, the tear and impression cytology samples were collected in the same manner. The expression of the *ACE2* gene was significantly higher in the conjunctiva of smokers (*n* = 17; median 3.07 copies/ng of total RNA) than in those of non-smokers (*n* = 17; median 1.92 copies/ng of total RNA, *p* = 0.003). Further, mRNA expression and protein levels of ACE2 were weakly correlated in smokers (r = 0.49). ACE2 protein levels in Schirmer’s strip samples were significantly reduced from 5051 to 3202 pg/mL after eye washing (*n* = 10; *p* = 0.001). Ocular surface cells are susceptible to SARS-CoV-2 infection. Smoking may be a risk factor for SARS-CoV-2 infection, and eye rinsing may reduce the risk of infection.

## 1. Introduction

Smoking is a risk factor for the occurrence of many respiratory infections and increases the severity of respiratory diseases. A review of studies conducted by public health experts convened by the World Health Organization (WHO) on 29 April 2020 found that smokers were more likely to develop severe coronavirus disease 2019 (COVID-19) than non-smokers [1]. The WHO also states that hand-to-mouth contact, smoking-induced lung disease, and sharing of tobacco products among smokers may increase their vulnerability to severe acute respiratory syndrome coronavirus 2 (SARS-CoV-2) infection and the development of COVID-19 [2].

COVID-19 is an infectious disease that primarily attacks the epithelial cells in the respiratory tracheal epithelium and other external organs, such as the ocular surface. Smoking impairs lung function, making it difficult for the body to fight off coronaviruses and other diseases. Tobacco is also a major risk factor for the occurrence of many non-communicable diseases, such as cardiovascular disease, cancer, respiratory disease, and diabetes, resulting in a higher risk of developing severe illness when infected with COVID-19. Therefore, smokers are at higher risk of developing severe disease and of death [3,4,5,6].

The WHO is constantly evaluating new research, including studies examining the links between tobacco and nicotine use and COVID-19. The WHO urges researchers and media to be cautious in amplifying unproven claims that tobacco or nicotine may reduce the risk of COVID-19. Currently, there is insufficient information to confirm a possible link between tobacco and nicotine in the prevention or treatment of COVID-19 [3,4].

Angiotensin-converting enzyme 2 (ACE2; the receptor for SARS-CoV-2) is expressed in various human organs, and its organ- and cell-specific expression suggests a role in the regulation of cardiovascular and renal function and fertility. In addition, the encoded protein is a functional receptor for the spike glycoprotein of the human coronavirus (HCoV-NL63), and human SARS-CoV strains SARS-CoV-1 and SARS-CoV-2, the latter of which is the causative agent of COVID-19. Analysis of surgical conjunctival specimens confirmed the expression of ACE2 and transmembrane protease serine 2 (TMPRSS2) in the conjunctival epithelium [5].

This study aimed to determine whether ocular surface cells produce key factors such as ACE2, TMPRSS2, and mucin 5AC (MUC5AC), which are required for cellular susceptibility to SARS-CoV-2 infection, and whether tobacco smoking affects the expression of ACE2 and TMPRSS2 on the ocular surface.

## 2. Results

### 2.1. mRNA Expression on the Ocular Surface

**Figure 1:**
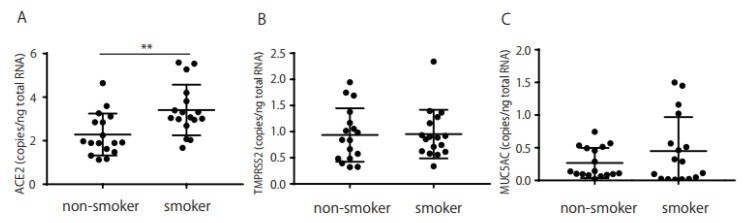
*ACE2*, *TMPRSS2*, and *MUC5AC* mRNA expression in smokers and non-smokers. *ACE2* mRNA expression was detected in conjunctival impression cytology (IC) samples and was significantly higher in smokers than in non-smokers (**A**); *p* < 0.01. *TMPRSS2* and *MUC5AC* mRNA were also detected but were not highly expressed in smokers (**B**,**C**). The horizontal bar represents the groups, while the vertical bar represents the mRNA copies/ng total RNA. *ACE2* mRNA is expressed in smokers. ** *p* < 0.01 was calculated using the Mann–Whitney U test.

RT-PCR was performed to determine whether the stored IC conjunctival samples expressed *ACE2, TMPRSS2*, or *MUC5AC*. *ACE2* expression was significantly higher in the conjunctiva of smokers (*n* = 17; median = 3.07 copies/ng of total RNA) than in non-smokers (*n* = 17; median 1.92 copies/ng of total RNA; *p* = 0.003, Figure 1A), whereas *TMPRSS2* and *MUC5AC* expression did not differ significantly between smokers (median = 0.89 copies/ng and 0.29 copies/ng of total RNA, respectively) and non-smokers (median 0.84 and 0.14 copies/ng of total RNA, Figure 1B and 1C, respectively).

### 2.2. ELISA of Schirmer’s Strip Samples

**Figure 2:**
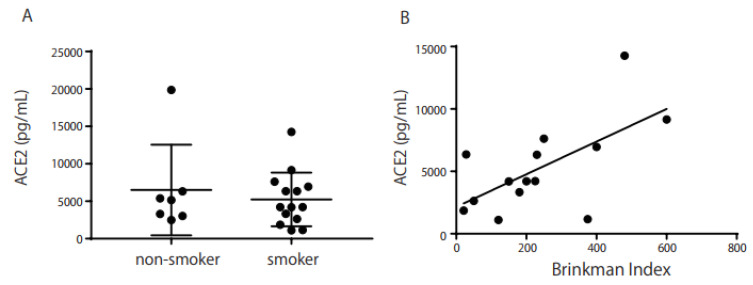
ACE2 production in Schirmer’s strip samples, and correlation between ACE2 and the Brinkman index (BI). ACE2 production was detected in Schirmer’s strip samples, but no significant difference was observed between smokers and non-smokers ((**A**) *p* = 0.34). A significant correlation was found between ACE2 in Schirmer’s strip samples and the BI ((**B**) r = 0.63, *p* = 0.02). The horizontal bar represents the BI score, whereas the vertical bar represents the ACE2 concentration in Schirmer’s strip samples (pg/mL). Statistical significance was calculated using the Mann–Whitney U test (**A**), and the correlation coefficient was analyzed using Spearman’s rank correlation (**B**).

**Figure 3:**
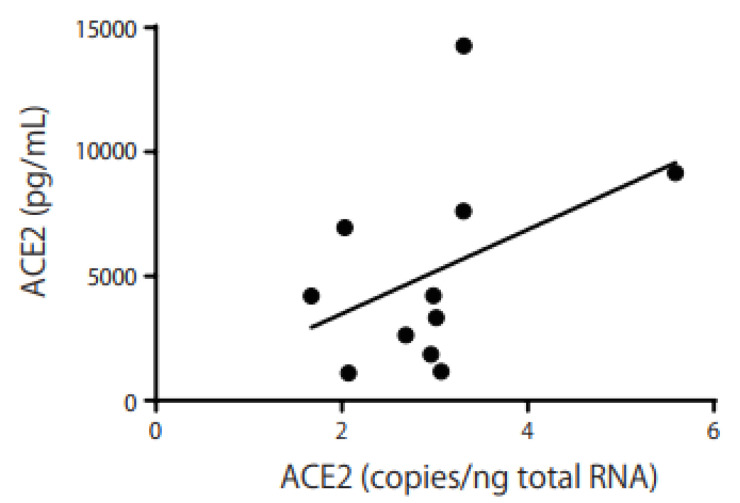
Correlation between *ACE2* mRNA and protein. A weak correlation was observed between the *ACE2* mRNA and protein levels (r = 0.43). The horizontal bar represents the *ACE2* mRNA copies/ng of total RNA, while the vertical bar represents the ACE2 protein concentration in Schirmer’s strip samples (pg/mL). The correlation coefficients were analyzed using Spearman’s rank correlation.

**Figure 4:**
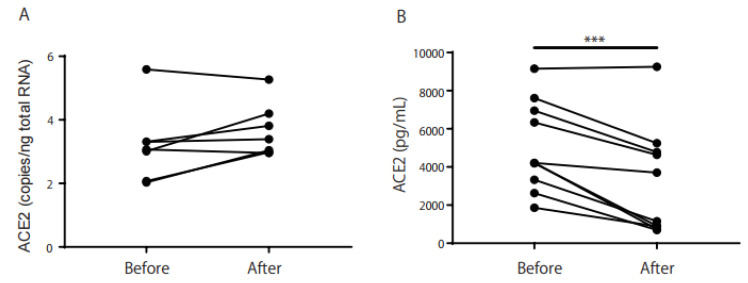
The washing effect on *ACE2* mRNA and protein production in Schirmer’s strip samples. *ACE2* mRNA expression was not significantly reduced through washing the eye for 30 s with a 5 min rest period ((**A**) *p* = 0.08), but the ACE2 protein levels in the Schirmer’s strip samples were significantly reduced ((**B**) *p* < 0.001). *** *p* < 0.001 was calculated using the paired *t*-test.

We found no significant increase in ACE2 levels in Schirmer’s strips of smokers (n = 14; median 4217 pg/mL) compared with non-smokers (*n* = 7; median 5163 pg/mL, Figure 2A). ACE2 expression in the Schirmer’s strips of smokers correlated with the Brinkman index (BI; r = 0.63, *p* = 0.02, Figure 2B). There was also a weak correlation between *ACE2* mRNA and protein levels in the Schirmer’s strips of smokers (r = 0.49, Figure 3). We used a commercially available wash solution for 5 min for individuals who smoked. *ACE2* mRNA expression increased slightly from 3.20 to 3.67 copies/ng of total RNA after eye washing (*n* = 7; *p* = 0.08, Figure 4A), whereas the ACE2 protein level in Schirmer’s strips decreased significantly from 5051 to 3202 pg/mL after eye washing (*n* = 10; *p* = 0.001, Figure 4B). *TMPRSS2* and *MUC5AC* mRNA and protein levels in Schirmer strips were not reduced through eye washing.

## 3. Discussion

We studied the expression of ACE2, TMPRSS2, and MUC5AC in ocular surface samples and Schirmer’s strips and investigated three key factors in SARS-CoV-2 infection in smokers and non-smokers. We also studied the effects of eye rinses on ACE2 expression and production. Roehrich et al. studied these factors in conjunctival IC samples and found that ACE2 expression and production were higher in smokers than in non-smokers and decreased after eye rinsing [7].

COVID-19 is caused by an emergent pathogenic coronavirus, SARS-CoV-2, which is phylogenetically similar to SARS-CoV-1, with approximately 80% similarity between their genomes [8]. All SARS viruses infect the respiratory tract and cause an acute respiratory response via the same cell entry receptor, ACE2, which is the only experimentally confirmed SARS-CoV-2 receptor. SARS-CoV-2 infection also activates the spike proteins found on the surface of the virus for cell entry. The best candidates for priming spike proteins are two host cell enzymes: furin and TMPRSS2 [8].

As shown in Figure 1, *ACE2* and *TMPRSS2* mRNAs were expressed in the conjunctival IC samples, and ACE2 protein was detected in Schirmer’s strips (Figure 2). These results imply that SARS-CoV-2 infection may occur via the ocular surface, similar to the way it occurs via the respiratory system. *ACE2* mRNA was significantly expressed in smokers (Figure 1), while ACE2 protein was detected in Schirmer’s strips (Figure 2A) and was correlated with BI in smokers (Figure 2B). In addition, a correlation was observed between *ACE2* mRNA and protein levels in the Schirmer’s strip samples (Figure 3). These results imply that ACE2 is highly expressed in smokers and may easily lead to SARS-CoV-2 infection and the subsequent development of COVID-19 via the ocular surface.

Salah et al. [9] and Grundy et al. [2] conducted meta-analyses and found a significant association between COVID-19 and smoking status. Ahmed et al. reported that tobacco smoking is a potential risk factor for SARS-CoV-2 transmission [10]. Liu et al. reported that cigarette smoke induces ACE2 overexpression in bronchial and alveolar epithelial cells [11]. Brake et al. presented evidence of ACE2 receptor expression in surgically resected lung tissues from smokers [5]. Lee et al. compared ACE2 expression between e-cigarette and non-e-cigarette users [3]. Lung and bronchial epithelial samples, particularly bronchial transient secretory cells, express significant amounts of ACE2 [12].

Our present findings confirm the results of earlier studies showing that smoking induces ACE2 expression and production and may allow for easier infection of SARS-CoV-2 via the ocular surface.

Recently, Arora et al. detected SARS-CoV-2 RNA in the tears of 24% of patients with laboratory-proven moderate-to-severe COVID-19 [13]. This finding suggests that SARS-CoV-2 is highly infectious in smokers owing to their high ACE2 expression, which can cause COVID-19. We studied the effects of eye-wash solutions on ACE2 expression and production. We found no significant reduction in *ACE2* mRNA expression after washing (Figure 4A). However, ACE2 protein levels in Schirmer’s strips were significantly reduced (Figure 4B). Because ACE2 and TMPRSS2 are membrane proteins, these results indicate that eye rinsing may contribute to the reduction in SARS-CoV-2 infection through flushing out ACE2. Normal membrane proteins are easily washed away, and ACE2 protein remains unchanged. For protein expression analysis, we determined the cell count in the samples collected using Schirmer’s strips, and ACE2 expression was normalized to the number of cells. In this study, we evaluated 8 mm wet test strips instead of counting the cells.

Voinsky et al. found that TMPRSS4 expression was elevated in the bronchial epithelial cells of current smokers compared with that in never-smokers, but no TMPRSS2 expression was found [14]. However, the results of the present study differ from these findings. Our results suggest that higher bronchial TMPRSS4 levels in smokers increase the risk of SARS-CoV-2 infection. In the future, we plan to investigate the effect of smoking on the ocular surface using a small acrylic resin box that releases TMPRSS4.

Eye-wash solutions are commercially available in Japan. Many of these agents are safe and beneficial for patients with cedar pollen allergies and other ocular diseases [15,16]. Rinsing eyes with this solution may help prevent SARS-CoV-2 infection [17]. To avoid stimulation via the sampling method, we first collected IC samples from the right eye without rinsing. The left eye was then rinsed with a commercial eye-wash solution for 30 s. After 5 min, Schirmer’s strips and IC samples were collected from the left eye in the same manner as that for the right eye. Initially, samples were collected from the same eye before and after washing. However, sampling-induced inflammation in the sampling area may have affected the results of this study. Therefore, the other eye was used for after-wash sampling. We collected right eye samples without washing and left eye samples after washing.

After rinsing the eyes with a commercial solution, ACE2 protein expression in smoker samples decreased and did not differ significantly from that in non-smokers. This result suggests that eye washing may decrease the possibility of SARS-CoV-2 infection via the eye and that washing the nose and mouth may also help protect individuals from SARS-CoV-2 infection. *MUC5AC* mRNA expression did not change after rinsing, indicating the safety of the wash solution for mucinous conditions on the ocular surface, as reported in previous studies [16].

Studies with larger cohorts and more comprehensive RNA sequencing technologies would yield more robust findings and provide more information on the effects of smoking on mRNA expression in conjunctival epithelial cells. The number of subjects in this study is small, and the method still needs to be improved. Further large-scale studies are needed to consider the number of years of smoking and to increase the number of subjects. The effects of smoking on disease risk may involve pathways other than viral entry pathways. Washing solutions may affect the presence of ACE2 and reduce the risk of viral infection. However, the effects of smoking on the immune system require further investigations. Further studies are needed to determine whether nicotine or nitric oxide has therapeutic potential in patients with COVID-19.

## 4. Materials and Methods

Written informed consent was obtained from all participants at the Tsurumi University School of Dental Medicine (Institutional Review Board #1814). All experiments were conducted according to the tenets of the Declaration of Helsinki. An interview was conducted to determine participants’ smoking history.

This prospective study included 20 eyes from 10 smokers (eight men and two women; mean age: 40.1 ± 13.2 years), and 18 eyes from nine healthy non-smokers (four men and five women; mean age: 42.9 ± 7.5 years) (as control) for reverse-transcription polymerase chain reaction (RT-PCR). This study also included 28 eyes from 14 smokers (13 men and 1 woman; mean age: 40.36 ± 13.2 years), and 16 eyes from eight healthy non-smokers (four men and four women; mean age 40.3 ± 7.5 years) (as control) for enzyme-linked immunosorbent assay (ELISA). The investigation was conducted at two centers: the Department of Ophthalmology, Tsurumi University School of Dental Medicine; and the International University of Health and Cornea Eye Center, Tokyo Dental College, Ichikawa Hospital. None of the patients had a history of Stevens–Johnson syndrome; chemical, thermal, or radiation injury; bacterial, viral, or other unclassified conjunctivitis, such as toxic conjunctivitis; or any ocular surgery that altered the ocular surface. In addition, none of the participants had a history of using drugs or contact lenses that could alter the ocular surface. Routine ophthalmic examinations included best-corrected visual acuity measurements, slit-lamp examinations, and anterior-segment photography. All patients underwent Schirmer’s strip sampling. Conjunctival impression cytology (IC) was performed to determine the expression of ACE2, TMPRSS2, and MUC5AC on the ocular surface.

Schirmer’s strip and IC samples were first collected from the right eye of each patient. The left eye was then rinsed with a commercial eye-wash solution for 30 s. After 5 min, the Schirmer’s strip and IC samples were collected from the left eye in the same manner.

### 4.1. IC Sample Collection and RT-PCR [6]

An EyePrim (Opia Technologies SAS, Paris, France) was used to collect IC samples from the temporal bulbar conjunctiva. EyePrim is a commercial device that standardizes the material and size of the membrane and includes a convenient holder for the user to acquire a sample [18,19]. The membrane was placed in 500 µL of ISOGEN II (Nippon gene: 311-07361) and stored at −80 °C. Total RNA was extracted from ISOGEN II according to the manufacturer’s instructions, with a final elution volume of 30 µL. The extracted RNA was stored at −80 °C prior to RT-PCR analysis (ABI PRISM 7000). The RNA extracted from all samples was tested for the presence of ACE2, TMPRSS2, and MUC5AC using RT-PCR with Platinum SYBR Green qPCR SuperMi-UDG (11733-046; Invitrogen, Waltham, MA, USA).

The IC samples were preserved in ISOGEN II (Nippon Gene, Tokyo, Japan) at −80 °C. Total RNA was extracted, cleaned, and treated with DNase, according to the manufacturer’s instructions. cDNA was synthesized using SuperScript II Reverse Transcriptase (Thermo Fisher Scientific, Waltham, MA, USA). The cDNA (5 ng) was amplified using a 25 µL final volume in the presence of 1 µL of “Assay by Design” oligonucleotides (ACE2, TMPRSS2, MUC5AC, and glyceraldehyde-3-phosphate dehydrogenase *(GAPDH*)). The test gene primer and probe sets were optimized for concentration, amplification efficiency, and co-amplification with the housekeeping gene (*GAPDH*) primer and probe sets. RT-qPCR was performed according to the manufacturer’s instructions (Applied Biosystems; Weiterstadt, Germany). The thermal profile consisted of 50 °C for 2 min, then 95 °C for 2 min, followed by 40 cycles of 94 °C for 15 s and 60 °C for 30 s. Real-time data were acquired and analyzed using Sequence Detection System Software (Applied Biosystems: Ver1.2 3f2) with the manual baseline and threshold parameters adjusted. The mRNA expression levels were normalized to the median expression level of *GAPDH*. The following primer sequences were used:

ACE2 Forward: 5′-AAACATACTGTGACCCCGCAT-3′, Reverse: 5′-CCAAGCCTCAGCATATTGAACA-3′;
TMPRSS2 Forward: 5′-AATCGGTGTGTTCGCCTCTAC-3′, Reverse: 5′-CGTAGTTCTCGTTCCAGTCGT-3′;
MUC5AC Forward: 5′-CAGGGCTGGTACACCTTGTC-3′, Reverse: 5′-ACGACATCTGCATCGATTGGA-3′; and
GAPDH Forward: 5′-GAA GGT GAA GGT CGG AGT C-3′, Reverse: 5′-GAA GAT GGT GAT GGG ATT TC-3′.

### 4.2. Schirmer’s Strip Sample Collection and ELISA

Schirmer’s strip samples were collected on Schirmer’s measurement strips (Schirmer Tear Production Measuring Strips; Showa Yakuhin Kako, Tokyo, Japan), using a previously described method [20]. To standardize the number of cells, the strips were removed when the paper memory was wetted to 8 mm. The strips were cut at 8 mm and immersed in 312 µL of 0.01 M phosphate-buffered saline (pH 7.2) with 0.5 M NaCl + Tween 20 for 24 h at 4 °C. After adding the buffer at the same dilution rate (40×), the paper was removed and stored at −80 °C until the assay was performed [21]. The Schirmer’s strip samples were analyzed for ACE2 using ELISA (AViVA Systems Biology ACE2 ELISA kit: OKCD07767), according to the manufacturer’s instructions; an appropriate dilution was established; and the concentrations are expressed as the mean ± standard deviation.

### 4.3. Statistical Analyses

The differences between the two groups were analyzed using the Mann–Whitney U test and paired *t*-test. The correlations were assessed using the Spearman’s rank correlation test. Statistical significance was set at *p* < 0.05. All statistical analyses were performed using GraphPad Prism 7.0 (GraphPad Software, San Diego, CA, USA).

## Data Availability

Data are contained within the article.
